# Targeting on Nrf2/Sesn2 Signaling to Rescue Cardiac Dysfunction during High-Fat Diet-Induced Obesity

**DOI:** 10.3390/cells11162614

**Published:** 2022-08-22

**Authors:** Meredith Krause-Hauch, Julia Fedorova, Linda Ines Zoungrana, Hao Wang, Mohammad Kasim Fatmi, Zehui Li, Migdalia Iglesias, Lily Slotabec, Ji Li

**Affiliations:** 1 Department of Surgery, Morsani College of Medicine, University of South Florida, Tampa, FL 33612, USA; 2 James A. Haley Veterans’ Hospital, Tampa, FL 33612, USA; 3 Department of Medical Engineering, College of Engineering and Morsani College of Medicine, Tampa, FL 33612, USA

**Keywords:** Sesn2, Nrf2, obesity, high-fat diet

## Abstract

Obesity is of concern to the population because it is known to cause inflammation and oxidative stress throughout the body, leading to patient predisposition for health conditions such as diabetes, hypertension, and some cancers. However, some proteins that are activated in times of oxidative stress may provide cytoprotective properties. In this study, we aim to gain further understanding of the interconnection between Nrf2 and Sesn2 during obesity-related stress and how this relationship can play a role in cardio-protection. Cardiomyocyte-specific Sesn2 knockout (cSesn2^−/−^) and Sesn2 overexpressed (tTa-tet-Sesn2) mice and their wildtype littermates (Sesn2*^flox/flox^* and tet-Sesn2, respectively) were assigned to either a normal chow (NC) or a high-fat (HF) diet to induce obesity. After 16 weeks of dietary intervention, heart function was evaluated via echocardiography and cardiac tissue was collected for analysis. Immunoblotting, histology, and ROS staining were completed. Human heart samples were obtained via the LifeLink Foundation and were also subjected to analysis. Overall, these results indicated that the overexpression of Sesn2 appears to have cardio-protective effects on the obese heart through the reduction of ROS and fibrosis present in the tissues and in cardiac function. These results were consistent for both mouse and human heart samples. In human samples, there was an increase in Sesn2 and Nrf2 expression in the obese patients’ LV tissue. However, there was no observable pattern of Sesn2/Nrf2 expression in mouse LV tissue samples. Further investigation into the link between the Sesn2/Nrf2 pathway and obesity-related oxidative stress is needed.

## 1. Introduction

Obesity is an increasing issue within the world’s population, with an estimated 13% of adults worldwide being diagnosed as obese in 2014 [1]. The United States, in particular, has an increased incidence of obesity with 40% of adult Americans being diagnosed as obese [2]. Obesity can cause an increased risk for a variety of medical conditions such as diabetes mellitus, hypertension, cancers, some musculoskeletal disorders, neurodegenerative diseases, and mental health conditions [3,4,5]. Further, many recent studies have indicated that obese patients experience an increased risk for COVID-19 and amplified disease severity in the current COVID-19 pandemic [2]. The surplus of adipose tissue that occurs from obesity leads to hypoxia, low-grade chronic inflammation, and oxidative stress, which in turn, increases patient predisposition for various health conditions [4,6].

Oxidative stress has been proposed to be a major component in obesity that leads to various comorbidities [7,8]. Oxidative stress occurs when there is an excess of reactive oxygen species (ROS) and free radicals present in cells [8]. This excess, directly or indirectly, leads to the damage of tissues or organs, which in turn causes conditions such as aging, neurodegenerative diseases, and cardiovascular disease [9,10]. Further, in conditions of increased oxidative stress, inflammatory responses are triggered [11]. This activation of inflammatory responses leads to a further increase in ROS production, exacerbating oxidative stress and leading to chronic inflammation [11,12]. In the presence of obesity, the state of chronic inflammation is associated with metabolic syndrome, which encompasses type 2 diabetes, insulin resistance, dyslipidemia, and hypertension [12,13].

Sestrin2 (Sesn2) is a stress-inducible protein that is activated in times of oxidative stress [14,15]. When active, Sesn2 functions in the inhibition of mammalian target of rapamycin complex 1 (mTORC1) and the suppression of ROS production [15]. Nuclear factor erythroid-2-related factor-2 (Nrf2) is a transcription factor that is responsible for the transcription of several antioxidant genes [16,17,18]. In basal conditions, Nrf2 is confined to the cytoplasm through binding to Kelch-like ECH-associated protein 1 (Keap1) [16,17,18]. However, during times of oxidative stress, Sesn2 upregulation triggers Keap1 to release Nrf2, allowing it to accumulate in the nucleus and induce the transcription of antioxidant genes, including *Sesn2*. The activation of Sesn2 due to oxidative stress creates a positive feedback loop with Nrf2, resulting in increased expression of both proteins [18]. Therefore, due to this positive feedback loop, it would be expected that Sesn2 and Nrf2 levels would be positively correlated.

While the Sesn2–Nrf2 signaling pathway is well studied, the effects that these proteins have on obesity-related oxidative stress-induced cardiac dysfunction are not fully understood. This study explores the relationship of Sesn2 and Nrf2 and how their interaction plays a role in obesity-related cardiac dysfunction.

## 2. Materials and Methods

### 2.1. Experimental Animals

Sesn2*^flox/flox^* and tet-Sesn2 mice were bred in our laboratory from a C57BL/6J background. Mice with knockout of the S*esn2* gene in cardiomyocytes (cSesn2^−/−^) were generated from the breeding of Sesn2*^flox/flox^* mice with transgenic mice. The transgenic mice have a *Cre* gene that is autosomally integrated and driven by the cardiac-specific alpha-myosin heavy chain promoter (aMHC). Transgenic Cre mice were purchased from The Jackson Laboratory (Bar Harbor, ME, USA).

**Figure 1:**
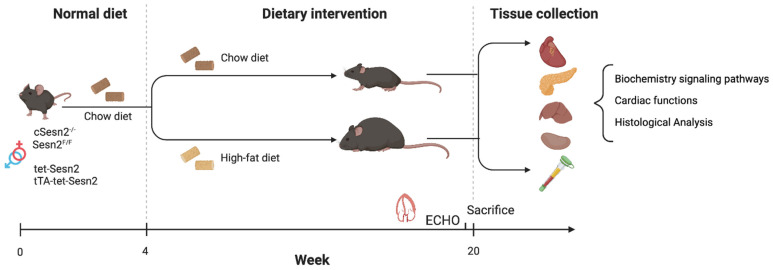
Experimental design and timeline. cSesn2^−/−^, tTa-tet-Sesn2, and their wildtype littermates (Sesn2*^flox^*^/*flox*^ and tet-Sesn2, respectively) were randomly assigned to a normal chow (NC) diet or a high-fat (HF) diet at 4 weeks of age. After 16 weeks of dietary intervention, echocardiogram data and tissue samples were collected for bioanalysis.

Mice (male and female) were randomly assigned to the normal chow (NC) group (control) or the high-fat (HF) chow group (*n* = 10–12 mice per strain/group). Mice in the NC group were fed a regular chow diet (17% energy from fat, 29% from protein, and 54% from carbohydrate; 300 kcal/100 g), while the HF chow group were fed a high-fat chow diet (54% energy from fat, 21% from protein, and 25% from carbohydrate; 490 kcal/100 g) (Catalog number TD.07011; Envingo Tekland Diets) for 16 weeks to induce obesity (Figure 1).

All mice were housed in a temperature-controlled environment on a typical 12 h light/dark cycle. Body weights were measured weekly to determine the rate of weight gain among the groups.

This study protocol was approved by the Institutional Animal Care and Use Committees of the University of South Florida.

### 2.2. Human Tissue Samples

Human heart samples were donated via the LifeLink Foundation. Upon receipt of organs, tissue samples were collected from each of the four chambers: left atrium, right atrium, left ventricle, and right ventricle. Samples were preserved in 4% paraformaldyde in phosphate-buffered saline (PBS), RNAlater solution, and embedded in O.C.T. embedding medium. Protein samples were stored at −80 °C for later testing. Along with the organ samples, Donor Summaries were provided by LifeLink. These summaries included additional information about the patient such as medical history, body weight and height, and testing results. From this information, the body mass index (BMI) was calculated to assess whether a patient could be included in one of two groups: ideal BMI (18.5–24.9) or obese BMI (≥30.0). BMI was calculated as BMI = weight (kg)/[height (m)]^2^.

### 2.3. Echocardiographic Evaluation

At the end of the study period (16 weeks of NC or HF diet), echocardiograms were performed on all mice to assess cardiac function. This was completed utilizing a Vevo 3100 imaging system (VisualSonics Inc., Toronto, ON, Canada) as previously described [19]. The following variables were assessed for all mice: left ventricular (LV) ejection fraction (LVEF), LV fractional shortening (LVFS), LV mass, the ratio of early diastolic-to-late diastolic mitral inflow velocities (E/A), and E-to-early diastolic mitral annular tissue velocity ratio (E/e′).

### 2.4. Histopathology

Both mouse and human samples underwent histopathological analysis to assess morphological changes under obesity. Upon collection, tissues were fixed in 4% paraformaldehyde in PBS. These were later embedded in paraffin and cut into 5 μm sections. These sections underwent hematoxylin and eosin (H & E) staining to evaluate the cellular morphology. Additional tissue sections were stained with Masson trichrome to assess the perivascular/interstitial fibrosis.

### 2.5. Western Blot Analysis

For both mouse and human samples, equal amounts of total protein from LV myocardial tissues were electrophoresed on 10% SDS-PAGE gels and transferred to polyvinylidene difluoride membranes. The membranes were incubated with primary antibodies overnight at 4 °C, washed, and then incubated with secondary antibodies for one hour. Immunoreactive bands were detected with SuperSignal West Femto (Thermo Fisher Scientific, Waltham, MA, USA) in a Western blotting detection system (Bio-Rad Laboratories, Hercules, CA, USA). Results are expressed as density values normalized to GAPDH.

### 2.6. Measurement of Mitochondrial Reactive Oxygen Species

The production of cardiac mitochondrial reactive oxygen species (ROS) was determined in both human LV tissues and mouse LV tissues using our previously described protocol [20]. Briefly, this was evaluated via wheat germ agglutinin (WGA) and MitoSOX Red staining. Fresh-frozen LV samples embedded in O.C.T. medium were cut to a 10 μm diameter for staining. Samples were washed with 1× PBS prior to being incubated in MitoSOX Red mitochondrial superoxide indicator (Invitrogen, Waltham, MA, USA) with 1× PBS at a 1:1000 dilution for 10 min at 37 °C, while avoiding light. Then, samples were washed again with 1× PBS and were incubated in WGA with 1× PBS at a 1:200 dilution for 10 min at 37 °C, while avoiding light. A fluorescence microscope was used for acquiring images (excitation: 510 nm; emissions: 580 nm). The fluorescence intensity by area was calculated via Fiji ImageJ Version 2.3.0 (ImageJ Software, Madison, WI, USA) using corrected total cell fluorescence formula and colocalization ratio by default colocalization threshold.

### 2.7. Statistical Analysis

All data are expressed as means ± SE, unless otherwise stated. Differences among more than two groups were compared by one- or two-way ANOVA for multiple comparisons and post hoc tests for selected comparisons. *p* < 0.05 was accepted as statistically significant. Statistical analyses were performed with Prism 8 (GraphPad Software, San Diego, CA, USA).

### 2.8. Data and Resource Availability

The data sets analyzed during the current study are available from the corresponding author on reasonable request.

## 3. Results

### 3.1. Weight Gain Variation in Cardiomyocyte Sesn2 Knockout and Overexpressed Mice

**Figure 2:**
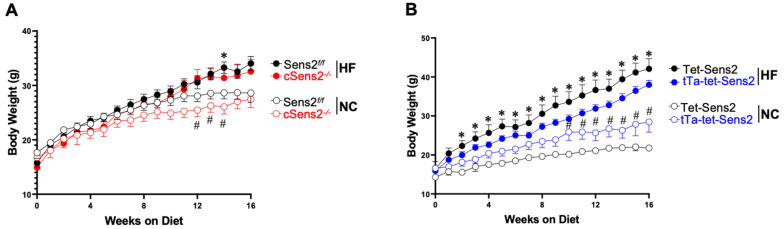
Body weight over time. Body weights were measured weekly until mice reached 16 weeks of dietary intervention. (**A**) Comparison of body weights over 16 weeks for Sesn2*^flox^*^/*flox*^ and cSesn2^−/−^ mice on NC and HF diets. * *p* < 0.05 vs. Sesn2*^flox^*^/*flox*^ NC; # *p* < 0.05 vs. cSesn2^−/−^ HF. (**B**) Comparison of body weights over 16 weeks for tet-Sesn2 and tTa-tet-Sesn2 mice on NC and HF diets. * *p* < 0.05 vs. tet-Sesn2 NC; # *p* < 0.05 vs. tTa-tet-Sesn2 HF. Data are means ± SE.

Over the 16 weeks of their selected diet, all groups had an increase in body weight (Figure 2A,B). However, as expected, mice that were fed the HF diet gained weight at a more rapid pace and, on average, weighed more at the experimental endpoint (weight at endpoint: Sesn2*^flox/flox^* NC: 28.6 ± 3.9 g; Sesn2*^flox/flox^* HF: 34.0 ± 3.4 g; cSesn2^−/−^ NC: 26.7 ± 5.5 g; cSesn2^−/−^ HF: 32.6 ± 5.9 g; tet-Sesn2 NC: 21.8 ± 0.9 g; tet-Sesn2 HF: 42.1 ± 5.8 g; tTa-tet-Sesn2 NC: 28.5 ± 6.4 g; tTa-tet-Sesn2 HF: 38.0 ± 3.9 g; mean ± SD). There was no significant difference between the weights of Sesn2*^flox/flox^* and cSesn2^−/−^ (*p* > 0.05; Figure 2A) or tet-Sesn2 and tTa-tet-Sesn2 (*p* > 0.05; Figure 2B) over the 16 weeks. However, each genotype group did show a significant difference between their NC and HF treatment groups (*p* < 0.05).

### 3.2. Relationship between Sesn2 and Nrf2 in Cardiomyocytes

**Figure 3:**
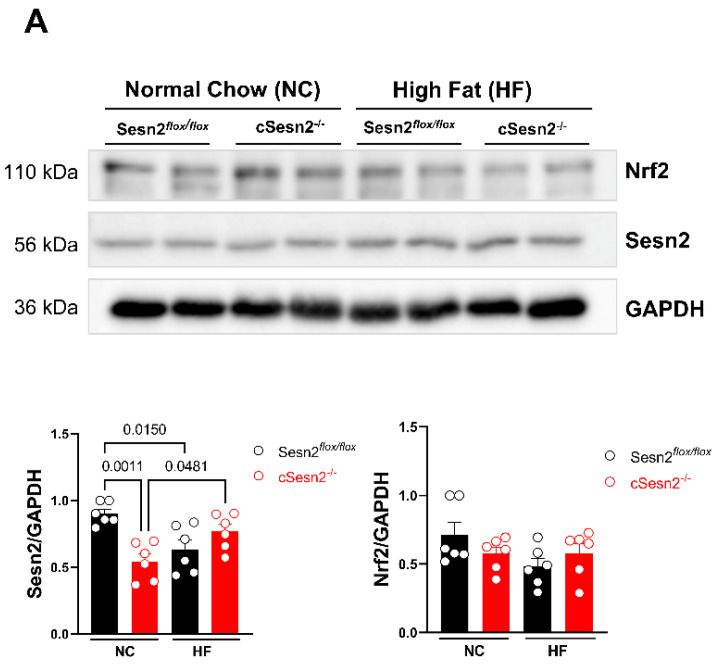

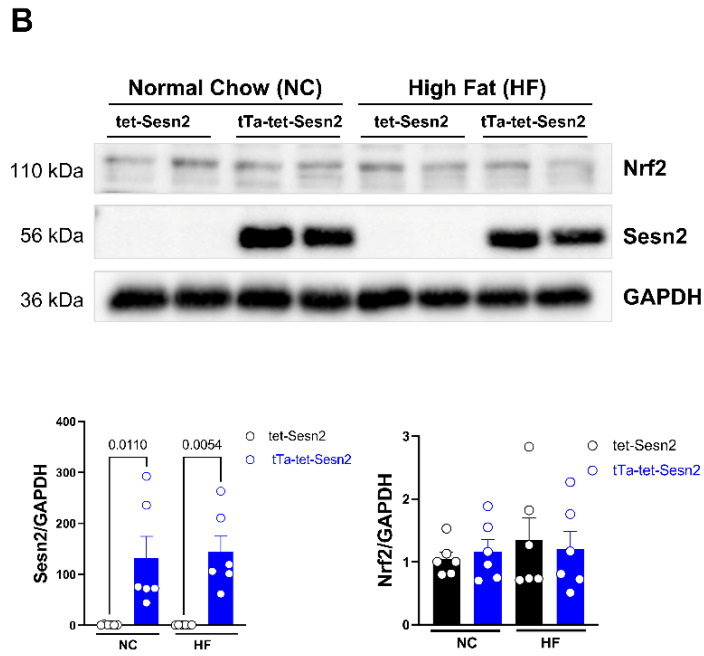
Western blotting results and representative images. Sesn2 and Nrf2 expression in (**A**) Sesn2*^flox^*^/*flox*^ and cSesn2^−/−^ and (**B**) tet-Sesn2 and tTa-tet-Sesn2 mice under NC and HF diets. Analysis is means ± SE. *n* = 6 per group.

In this study, it was expected that Sesn2 and Nrf2 would exhibit similar levels across groups and treatments due to their positive feedback loop that occurs during times of oxidative stress. In cSesn2^−/−^ mice, there was a significant increase in Sesn2 expression from NC- to HF-fed mice (*p* < 0.05; Figure 3A). However, there was no difference in the expression level of Nrf2 between cSesn2^−/−^ NC and HF mice. A similar trend of expression levels of Sesn2 and Nrf2 was observed in Sesn2*^flox/flox^*; HF mice had less expression of both proteins compared to NC mice. Due to the overexpression of Sesn2 in cardiomyocyte tissue, tTa-tet-Sesn2 mice expressed significantly more Sesn2 than their wildtype littermates did (tet-Sesn2; Figure 3B). However, there was no difference in the level of Sesn2 and Nrf2 between NC and HF tTa-tet-Sesn2 mice. There was a mild increase in Nrf2 expression from NC to HF in tet-Sesn2 mice.

### 3.3. Sesn2 Preserves Cardiac Function in Obese Mice

**Figure 4:**
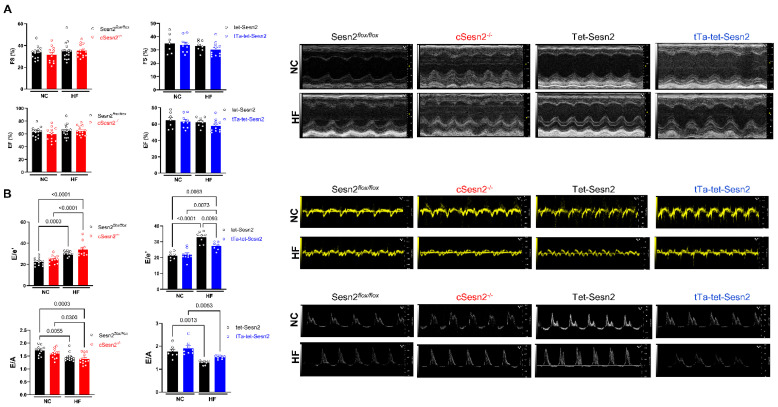
Effect of Sesn2 on cardiac function after 16 weeks of dietary intervention. (**A**) Systolic function of the heart (LVFS and LVEF). (**B**) Diastolic function of the heart (E/A and E/e′). *n* = 6–8 per group. Means ± SE.

To test the cardiac function of the mice, an echocardiogram was performed on each mouse after 16 weeks on their assigned diet (Figure 4). There were no differences in systolic function (LVEF and LVFS) across all groups (Figure 4A). However, diastolic function (E/A and E/e′) was impaired in mice that consumed the HF diet. Further, greater diastolic impairment was observed in cSesn2^−/−^ mice on the HF diet, while diastolic function was more preserved in HF tTa-tet-Sesn2 mice (Figure 4B).

### 3.4. The Overexpression of Sesn2 Provided Cardiac Protection from Fibrosis

**Figure 5:**
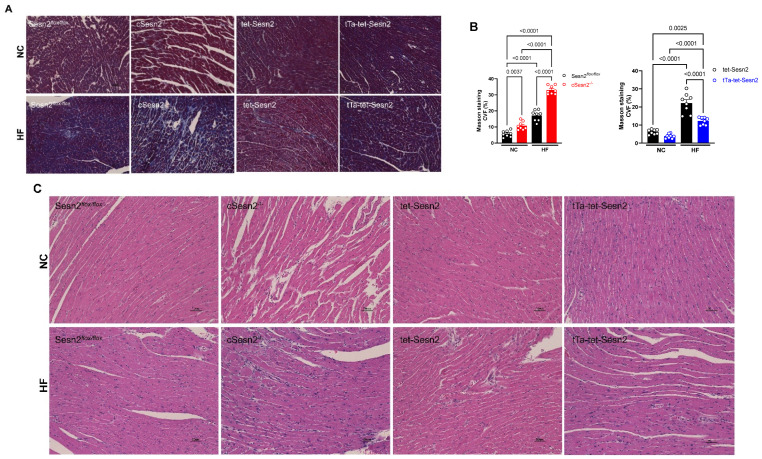
Effect of Sesn2 on the accumulation of cardiac fibrosis and inflammatory damage. (**A**) Representative Masson’s trichrome staining. (**B**) Collagen volume fraction (CVF). Means ± SE. (**C**) Representative hematoxylin and eosin staining; *n* = 8 per group.

Mice fed a HF diet for 16 weeks had significantly (*p* < 0.05) more fibrosis present in their heart tissue compared to mice fed a normal chow diet (Figure 5A,B). However, cSesn2^−/−^ mice fed a HF diet developed significantly more fibrotic tissue than their wildtype littermates did (Sesn2*^flox/flox^*; *p* < 0.05). Conversely, significantly less fibrotic tissue was observed in tTa-tet-Sesn2 HF mice than in their wildtype littermates (tet-Sesn2) fed the same diet (*p* < 0.05). Increased evidence of inflammation and cellular damage was observed in HF diet-fed mice (Figure 5C). cSesn2^−/−^ HF diet mice had greater cellular morphology changes compared to their wildtype counterparts (Sesn2*^flox/flox^*). The overexpression of Sesn2 present in tTa-tet-Sesn2 appears to have provided some cardiac protection from cellular damage and inflammation under HF diet-induced obesity.

### 3.5. ROS Presence in Obese Mice Cardiac Tissue

**Figure 6:**
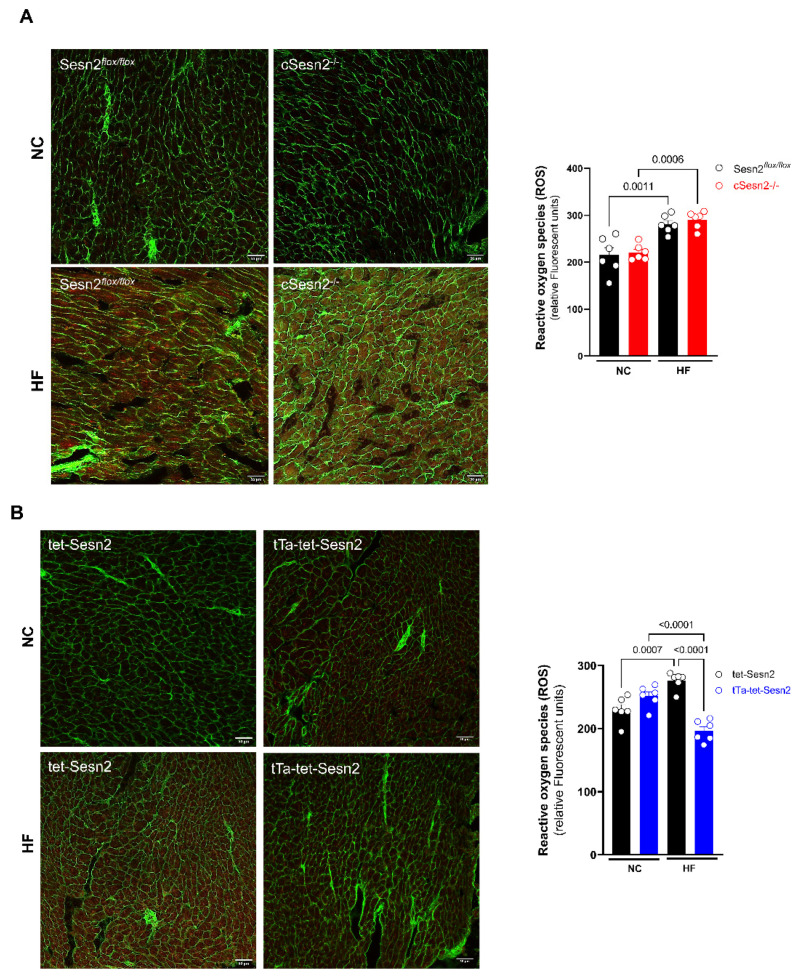
ROS staining results and representative images for mouse LV tissue. (**A**) Sesn2^f*lox*/*flox*^ and cSesn2^−/−^ and (**B**) tet-Sesn2 and tTa-tet-Sesn2. Means ± SE. *n* = 6 per group.

ROS presence in Sesn2 cardiomyocyte-specific knockout and overexpressed mice with HF diet-induced obesity was evaluated in order to determine the level of oxidative stress occurring in LV tissue. There was no difference in the levels of ROS present in Sesn2*^flox/flox^* and cSesn2^−/−^ tissue for either NC or HF diet mice. However, both Sesn2*^flox/flox^* and cSesn2^−/−^ mice had significantly more ROS present in mice fed a HF diet compared to their NC counterparts (Figure 6A). Additionally, there was no difference in ROS expression between tet-Sesn2 and tTa-tet-Sesn2 mice following an NC diet (Figure 6B). There was a significant increase in the levels of ROS in HF diet-fed tet-Sesn2 mice in comparison to those fed an NC diet. Interestingly, HF diet-fed tTa-tet-Sesn2 mice had significantly less ROS production compared to those fed an NC diet.

### 3.6. Effects of Obesity on Human Cardiac Tissue

**Figure 7:**
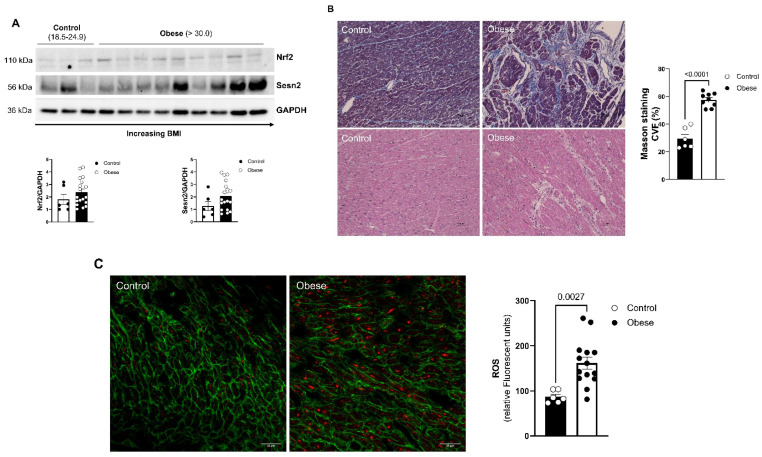
Effect of obesity on human hearts. (**A**) Western blot analysis of Sesn2 and Nrf2 in human LV tissue. (**B**) Representative Masson’s trichrome and hematoxylin and eosin staining; CVF = collagen volume fraction. (**C**) Representative images of mitochondrial superoxide production (MitoSox). Normal BMI (control) *n* = 3; obese BMI (obese) *n* = 9. Means ± SE.

Approximately 41% of hearts that were received from donors were from obese patients (BMI > 30.0). Of these samples, all patients that were >65 years old and who already had a history of significant heart disease were excluded. This resulted in *n* = 9 obese patients and *n* = 3 patients with ideal BMI (BMI = 18.5–24.9) for testing. The average BMI of patients with an ideal BMI was 21.0, while the average BMI for obese patients was 36.7. Human hearts showed similar levels of Nrf2 and Sesn2 expression (Figure 7A). Obese patients exhibited greater levels of both Sesn2 and Nrf2, with lower levels of expression in patients that were an ideal BMI. The amount of fibrotic tissue present in ideal-BMI hearts and obese-BMI hearts was evaluated using trichrome staining. Obese patients were revealed to have significantly more fibrosis present in LV tissue compared to patients with an ideal BMI (Figure 7B). To verify that the hearts of obese patients exhibit a greater amount of oxidative stress, MitoSOX/WGA staining was completed to determine the level of ROS occurring in these tissues. There was a significant increase in the presence of ROS from patients with normal BMI to obese patients, indicating that hearts in obese patients undergo more oxidative stress conditions compared to hearts in patients with ideal BMIs (Figure 7C).

## 4. Discussion

The experiments described above indicate that Sesn2 overexpression exhibits cardioprotective properties under obesity-related stress. Here, mice fed a HF chow diet and with an overexpression of Sesn2 (tTa-tet-Sesn2) present in their cardiomyocytes were more protected from weight gain compared to their wildtype littermates (tet-Sesn2), Sesn2*^flox/flox^*, and cSesn2^−/−^ mice (Figure 2). Additionally, HF diet-fed tTa-tet-Sesn2 mice were observed to have a diastolic function that was significantly improved from wildtype littermates that were fed the same HF diet. Further, these mice experienced significantly less ROS and fibrosis in LV heart tissue. Mice with a knockout of Sesn2 in their cardiomyocytes (cSesn2^−/−^) observed opposite trends, with HF-fed mice experiencing a decline in diastolic function and increased ROS and fibrotic tissue. Overall, this study demonstrates a cardioprotective role of Sesn2 under obesity-related stress.

While we were able to confirm that Sesn2 does provide protection to cardiac function under obesity-related stress, our results do not indicate any clear pattern for the up- or downregulation of Sesn2 or Nrf2 under these conditions. Sesn2*^flox/flox^* and cSesn2^−/−^ mice showed opposite patterns of Sesn2 expression, with HF-fed Sesn2*^flox/flox^* mice exhibiting significant downregulation of Sesn2 compared to their NC-fed counterparts. Conversely, HF-fed cSesn2^−/−^ mice showed significant Sesn2 upregulation in comparison to NC-fed cSesn2^−/−^ mice. While cSesn2^−/−^ mice have a knockout of Sesn2, this knockout only occurs in cardiomyocytes. Sesn2 is still present within other cardiac cell types such as smooth muscle cells, endothelial cells, and fibroblasts. It is likely that the Sesn2 expression that is observed is due to levels present in other cell types. It would be beneficial for any subsequent studies to isolate cardiomyocytes prior to immunoblotting. Additionally, further investigation using a global Sesn2 knockout/overexpression may provide a clearer pattern of protein expression. No significant difference in Nrf2 expression between NC- or HF-fed Sesn2*^flox/flox^* or cSesn2^−/−^ mice was observed. However, a similar pattern of Nrf2 expression is seen in the Sesn2*^flox/flox^* mice. Further, there was no significant difference in Sesn2 expression between NC and HF tet-Sesn2 or tTa-tet-Sesn2 mice. Similarly, there was no observed significant difference between NC or HF tet-Sesn2 or tTa-tet-Sesn2. Although, we do see an opposite pattern occurring between Sesn2*^flox/flox^* mice and tet-Sesn2 mice—tet-Sesn2 has a mild upregulation of Nrf2 under a HF diet while Sesn2*^flox/flox^* has a mild downregulation of Nrf2 under the same condition. While these results do not indicate any definitive pattern for the expression of Sesn2 and Nrf2 in the LV of HF diet-induced obese mice, other studies have found a correlation between these two proteins. Wang et al. observed an increase in Nrf2 in the brain when there was an overexpression of Sesn2 [21], while Fan et al. showed that Sesn2 protected retinal ganglion cells via activation of Nrf2 activity [16]. However, Zhang et al. (2021) observed that Sesn2 was upregulated for the first 4 weeks post aortic binding surgery, but was then downregulated to below baseline values at 8 weeks post-surgery [22]. When Nrf2 is activated, it is translocated from the cytoplasm to the nucleus [23]. This change in location may be the reason we do not observe a clear pattern of expression for Nrf2 or for the relationship between Sesn2 and Nrf2. Once inside the nucleus, Nrf2 is known to bind to the antioxidant response element (ARE) promoter region for *Sesn2*, stimulating the transcription of *Sesn2* [24,25]. The promotion of *Sesn2* transcription indicates that as more Nrf2 is activated, more Sesn2 should also become activated. Moreover, the activation of Sesn2 due to oxidative stress has previously been shown to degrade Keap1, allowing for the translocation of Nrf2 to the nucleus [18,26]. This suggests that a positive feedback loop occurs between Sesn2 and Nrf2 in the presence of oxidative stress. Additional methods such as testing for Nrf2 nuclear translocation, the transcription of Nrf2 target genes, or cell culture model systems could clarify Nrf2 levels and indicate a distinct pattern of expression with Sesn2. Further investigation into how Sesn2 and Nrf2 expression changes over time during obesity could provide insight into how these proteins interact over time during obesity-related stress conditions.

Cardiac fibrosis occurs when there is an excess of extracellular matrix proteins present in the myocardium, typically as a result of chronic activation of inflammatory pathways [27,28,29]. Other conditions related to obesity that can lead to cardiac fibrosis include hyperglycemia, insulin resistance, and metabolic dysfunction [30]. During times of cardiac stress (that may occur in obese patients), factors such as damage-associated molecular patterns (DAMPs) induce the secretion of inflammatory cells such as neutrophils and monocytes [31]. If these pathways are not shut off, resolving the inflammation, this leads to the chronic activation of fibroblasts and, thus, fibrosis [31]. Many obese patients have been shown to develop fibrosis of the myocardium [27,29]. Further, these fibrotic changes have been found to be connected to ventricular stiffness and diastolic dysfunction [29,30,32]. Here, we observed that obese mice and humans experienced greater amounts of fibrosis in tissue of the left ventricle compared to their counterparts that were of a more ideal weight or BMI. Additionally, significantly more fibrosis was observed in mice with a knockout of cardiomyocyte Sesn2, and less fibrosis was observed in those with an overexpression of cardiomyocyte Sesn2. While it is widely recognized that Sesn2 protects the heart from fibrosis, the pathway in which this occurs is currently unknown [14]. The results presented in this study appear to indicate that the Sesn2–Nrf2 pathway is not responsible for this protective effect. The physiological effects of the reduced levels of fibrosis can be observed in our echo data. Here, we see no significant differences in LVEF and LVES between mice on a NC diet and those on a HF diet. However, all groups experienced a decrease in E/A in HF mice. This reduction in E/A was moderately mitigated in mice with Sesn2 overexpression, compared to their wildtype littermates (tet-Sesn2), while E/A observed in Sesn2 knockout mice was reduced. In addition to fibrosis posing a challenge to cardiac function in obese individuals, the surplus of adipose tissue that is present around organs requires greater cardiac output, resulting in left ventricle hypertrophy [33,34].

Previous studies have indicated that under oxidative stress conditions, Sesn2 is upregulated [18]. The upregulation of this protein leads to pathways such as the Sesn2–Nrf2 pathway being activated. The Sesn2–Nrf2 pathway is of interest because it directly has an effect on the upregulation of various antioxidant enzymes and the suppression of ROS, among other effects [18]. Other Sesn2 signaling pathways such as Sesn2–AMPK and Sesn2–mTOR also play roles in the mitigation of inflammation and oxidative stress [18,35]. Previous studies have indicated that when Sesn2 is depleted in the heart, fatty acids are accumulated and ROS production increases [35]. In this study, the level of ROS present in obese patients’ LV samples was significantly greater than in those of patients with an ideal BMI. This increased level of ROS present in the heart indicates that there was more oxidative stress occurring in the cardiac tissue of obese patients (Figure 7C). Similar results were overserved in Sesn2*^flox/flox^*, cSesn2^−/−^, and tet-Sesn2 mice; greater amounts of ROS were present in the LV of those fed a HF diet than in the LV of those fed an NC diet (Figure 6). However, our results show a significant decrease in ROS presence in tTa-tet-Sesn2 HF diet-fed mice when compared to those fed an NC diet (Figure 6B). Sesn2 is known to reduce the accumulation of ROS in cells [22,36]. Based on our data presented here, it appears that the overexpression of Sesn2 provides extensive protective effects against ROS under oxidative stress conditions, thus attenuating the production of fibrosis in cardiac tissue.

There are some limitations of the human heart data that should be addressed. First, unlike in mouse models where mice are raised in a laboratory environment under controlled conditions, humans have a large variability in various aspects of their lives, such as in their diets and exercise regimens. Additionally, components such as race, socioeconomic status, and genetics can play a large role in the health status of individuals. Further, many of the human heart specimens that are donated to this lab are donated due to the heart being deemed unacceptable for transplantation. This status may be due to preexisting heart disease or may be due to a history of drug or alcohol use in the patient. Due to these reasons, it is difficult to control for so many variables when conducting studies on human samples. For instance, when performing Western blotting, it would be expected that Sesn2 and Nrf2 would become increasing upregulated as the BMI of the patient increased. However, our data show that in some patients with a lower BMI, Sesn2 and Nrf2 are expressed more, when compared to patients with a greater BMI (Figure 7A). This could be due to other co-existing health conditions, differences in diet, or variation in smoking, drug use, or drinking habits.

These results indicate that some mechanism involving Sesn2 is occurring that may reduce the activity of fibroblasts and thus pro-inflammatory cells in the heart as a response to the inflammation that results from obesity. Additional investigation into the levels of pro-inflammatory cells present in the company of Sesn2 deficiency or overexpression may elucidate further understanding into the mechanism in which Sesn2 protects cardiac tissue from fibrotic changes during obesity-induced chronic inflammation. In addition to causing inflammation and oxidative stress, obesity also causes mitochondrial dysfunction [37]. Mitochondrial dysfunction occurs when the mitochondria are unable to maintain adequate levels of ATP [37]. Previous studies have indicated that obesity and mitochondrial dysfunction are intertwined [37,38]. Further investigation into the interplay of Sesn2 with obesity and mitochondrial dysfunction could provide more insight into the mechanisms by which Sesn2 may provide cytoprotective properties. Even though much is already known about the relationship between Sesn2 and Nrf2, more knowledge is needed in order to fully understand how these two proteins may play a role in cardio-protection under obesity-related stress conditions.

## Data Availability

Not applicable.

## References

[1] Badimon L., Bugiardini R., Cenko E., Cubedo J., Dorobantu M., Duncker D.J., Estruch R., Milicic D., Tousoulis D., Vasiljevic Z. (2017). Position Paper of the European Society of Cardiology–Working Group of Coronary Pathophysiology and Microcirculation: Obesity and Heart Disease. Eur. Heart J..

[2] Kwok S., Adam S., Ho J.H., Iqbal Z., Turkington P., Razvi S., Le Roux C.W., Soran H., Syed A.A. (2020). Obesity: A Critical Risk Factor in the COVID-19 Pandemic. Clin. Obes..

[3] Chooi Y.C., Ding C., Magkos F. (2019). The Epidemiology of Obesity. Metabolism.

[4] Karczewski J., Śledzińska E., Baturo A., Jończyk I., Maleszko A., Maleszko A., Samborski P., Begier-Krasińska B., Dobrowolska A. (2018). Obesity and Inflammation. Eur. Cytokine Netw..

[5] Tarantini S., Valcarcel-Ares M.N., Yabluchanskiy A., Tucsek Z., Hertelendy P., Kiss T., Gautam T., Zhang X.A., Sonntag W.E., de Cabo R. (2018). Nrf2 Deficiency Exacerbates Obesity-Induced Oxidative Stress, Neurovascular Dysfunction, Blood–Brain Barrier Disruption, Neuroinflammation, Amyloidogenic Gene Expression, and Cognitive Decline in Mice, Mimicking the Aging Phenotype. J. Gerontol. Ser. A.

[6] Jimoh A., Tanko Y., Ahmed A., Mohammed A., Ayo J.O. (2018). Resveratrol Prevents High-Fat Diet-Induced Obesity and Oxidative Stress in Rabbits. Pathophysiology.

[7] Bondia-Pons I., Ryan L., Martinez J.A. (2012). Oxidative Stress and Inflammation Interactions in Human Obesity. J. Physiol. Biochem..

[8] Vincent H.K., Innes K.E., Vincent K.R. (2007). Oxidative Stress and Potential Interventions to Reduce Oxidative Stress in Overweight and Obesity. Diabetes Obes. Metab..

[9] Fernández-Sánchez A., Madrigal-Santillán E., Bautista M., Esquivel-Soto J., Morales-González Á., Esquivel-Chirino C., Durante-Montiel I., Sánchez-Rivera G., Valadez-Vega C., Morales-González J.A. (2011). Inflammation, Oxidative Stress, and Obesity. Int. J. Mol. Sci..

[10] Pan C., Chen Z., Li C., Han T., Liu H., Wang X. (2021). Sestrin2 as a Gatekeeper of Cellular Homeostasis: Physiological Effects for the Regulation of Hypoxia-Related Diseases. J. Cell. Mol. Med..

[11] Chatterjee S., Dziubla T., Butterfield D.A. (2016). Chapter Two—Oxidative Stress, Inflammation, and Disease. Oxidative Stress and Biomaterials.

[12] Verdile G., Keane K.N., Cruzat V.F., Medic S., Sabale M., Rowles J., Wijesekara N., Martins R.N., Fraser P.E., Newsholme P. (2015). Inflammation and Oxidative Stress: The Molecular Connectivity between Insulin Resistance, Obesity, and Alzheimer’s Disease. Mediat. Inflamm..

[13] Eckel R.H., Grundy S.M., Zimmet P.Z. (2005). The Metabolic Syndrome. Lancet.

[14] Wang(b) J., Wang S., Xiao M., Zhang J., Wang(a) J., Guo Y., Tang Y., Gu J. (2021). Regulatory Mechanisms of Sesn2 and Its Role in Multi-Organ Diseases. Pharmacol. Res..

[15] Sun W., Wang Y., Zheng Y., Quan N. (2020). The Emerging Role of Sestrin2 in Cell Metabolism, and Cardiovascular and Age-Related Diseases. Aging Dis..

[16] Fan Y., Xing Y., Xiong L., Wang J. (2020). Sestrin2 Overexpression Alleviates Hydrogen Peroxide-Induced Apoptosis and Oxidative Stress in Retinal Ganglion Cells by Enhancing Nrf2 Activation via Keap1 Downregulation. Chem. Biol. Interact..

[17] Park H.-J., Yang S.-G., Koo D.-B. (2022). SESN2/NRF2 Signaling Activates as a Direct Downstream Regulator of the PERK Pathway against Endoplasmic Reticulum Stress to Improve the in Vitro Maturation of Porcine Oocytes. Free. Radic. Biol. Med..

[18] Liu Y., Li M., Du X., Huang Z., Quan N. (2021). Sestrin 2, a Potential Star of Antioxidant Stress in Cardiovascular Diseases. Free. Radic. Biol. Med..

[19] Wang L., Quan N., Sun W., Chen X., Cates C., Rousselle T., Zhou X., Zhao X., Li J. (2018). Cardiomyocyte-Specific Deletion of Sirt1 Gene Sensitizes Myocardium to Ischaemia and Reperfusion Injury. Cardiovasc. Res..

[20] Murphy J., Le T.N.V., Fedorova J., Yang Y., Krause-Hauch M., Davitt K., Zoungrana L.I., Fatmi M.K., Lesnefsky E.J., Li J. (2022). The Cardiac Dysfunction Caused by Metabolic Alterations in Alzheimer’s Disease. Front. Cardiovasc. Med..

[21] Wang P., Zhao Y., Li Y., Wu J., Yu S., Zhu J., Li L., Zhao Y. (2019). Sestrin2 Overexpression Attenuates Focal Cerebral Ischemic Injury in Rat by Increasing Nrf2/HO-1 Pathway-Mediated Angiogenesis. Neuroscience.

[22] Zhang N., Liao H.-H., Feng H., Mou S.-Q., Li W.-J., Aiyasiding X., Lin Z., Ding W., Zhou Z.-Y., Yan H. (2021). Knockout of AMPKα2 Blocked the Protection of Sestrin2 Overexpression Against Cardiac Hypertrophy Induced by Pressure Overload. Front. Pharmacol..

[23] Deshmukh P., Unni S., Krishnappa G., Padmanabhan B. (2017). The Keap1–Nrf2 Pathway: Promising Therapeutic Target to Counteract ROS-Mediated Damage in Cancers and Neurodegenerative Diseases. Biophys. Rev..

[24] Zoungrana L.I., Krause-Hauch M., Wang H., Fatmi M.K., Bates L., Li Z., Kulkarni P., Ren D., Li J. (2022). The Interaction of MTOR and Nrf2 in Neurogenesis and Its Implication in Neurodegenerative Diseases. Cells.

[25] Shin B.Y., Jin S.H., Cho I.J., Ki S.H. (2012). Nrf2-ARE Pathway Regulates Induction of Sestrin-2 Expression. Free. Radic. Biol. Med..

[26] Bae S.H., Sung S.H., Oh S.Y., Lim J.M., Lee S.K., Park Y.N., Lee H.E., Kang D., Rhee S.G. (2013). Sestrins Activate Nrf2 by Promoting P62-Dependent Autophagic Degradation of Keap1 and Prevent Oxidative Liver Damage. Cell Metab..

[27] Frangogiannis N.G. (2021). Cardiac Fibrosis. Cardiovasc. Res..

[28] Wynn T. (2008). Cellular and Molecular Mechanisms of Fibrosis. J. Pathol..

[29] Dobaczewski M., Frangogiannis N.G. (2009). Chemokines and Cardiac Fibrosis. Front. Biosci..

[30] Cavalera M., Wang J., Frangogiannis N.G. (2014). Obesity, Metabolic Dysfunction, and Cardiac Fibrosis: Pathophysiological Pathways, Molecular Mechanisms, and Therapeutic Opportunities. Transl. Res..

[31] Felisbino M.B., McKinsey T.A. (2018). Epigenetics in Cardiac Fibrosis. JACC Basic Transl. Sci..

[32] Ren D., Fedorova J., Davitt K., Le T.N.V., Griffen J.H., Liaw P.C., Esmon C.T., Rezaie A.R., Li J. (2022). Activated Protein C Strengthens Cardiac Tolerance to Ischemic Insults in Aging | Circulation Research. Circ. Res..

[33] Aurigemma G.P., de Simone G., Fitzgibbons T.P. (2013). Cardiac Remodeling in Obesity. Circ. Cardiovasc. Imaging.

[34] Sun X., Pan H., Tan H., Yu Y. (2012). High Free Fatty Acids Level Related with Cardiac Dysfunction in Obese Rats. Diabetes Res. Clin. Pract..

[35] Liu Y., Li M., Sun M., Zhang Y., Li X., Sun W., Quan N. (2021). Sestrin2 Is an Endogenous Antioxidant That Improves Contractile Function in the Heart during Exposure to Ischemia and Reperfusion Stress. Free. Radic. Biol. Med..

[36] Kim K.M., Yang J.H., Shin S.M., Cho I.J., Ki S.H. (2015). Sestrin2: A Promising Therapeutic Target for Liver Diseases. Biol. Pharm. Bull..

[37] De Mello A.H., Costa A.B., Engel J.D.G., Rezin G.T. (2018). Mitochondrial Dysfunction in Obesity. Life Sci..

[38] Zorzano A., Liesa M., Palacín M. (2009). Role of Mitochondrial Dynamics Proteins in the Pathophysiology of Obesity and Type 2 Diabetes. Int. J. Biochem. Cell Biol..

